# Supplementary material for: Frontal and parietal planning signals encode adapted motor commands when learning to control a brain–computer interface
Source: PLoS Biol. 2025 Sep 29;23(9):e3003408. doi: 10.1371/journal.pbio.3003408 (PMC12533969; doi:10.1371/journal.pbio.3003408)
Supplement: S1 Table — Experimental design. This table summarizes, for each recording session, the number of units for each population, the type of decoder used, the rotation angle, and the direction of rotation (clockwise, CW, or counterclockwise, CCW). (PDF) [file pbio.3003408.s001.pdf]

file_name	M1_PMD_C	M1_PMD_NC	PRR_C	PRR_NC	Rotation_angle	Monkey	Decoder	Rotation_type
7/6/2016	44	12		37	30	Y	FO	CCW
7/8/2016	38	13		30	30	Y	FO	CCW
7/27/2016	45	6		48	30	Y	FO	CCW
7/28/2016	48	16		51	30	Y	FO	CCW
8/5/2016	40	24		39	30	Y	FO	CCW
8/9/2016	42	26		38	30	Y	FO	CCW
8/10/2016	36	25		32	30	Y	FO	CCW
8/13/2016	30	27		42	30	Y	FO	CCW
10/7/2016	43	10		28	30	Y	FO	CCW
10/12/2016	41	23		28	-30	Y	FO	CW
10/27/2016	42	8		23	-30	Y	FO	CW
11/2/2016	38	13		18	-30	Y	FO	CW
11/3/2016	35	18		23	-30	Y	FO	CW
11/4/2016	30	20		30	30	Y	FO	CCW
11/9/2016	30	16		22	30	Y	FO	CCW
12/7/2016	43	12		13	30	Y	FO	CCW
12/9/2016	41	19		19	30	Y	FO	CCW
12/20/2016	48	12		15	-30	Y	FO	CW
12/21/2016	38	14		20	30	Y	FO	CCW
1/4/2017	44	16		13	-30	Y	FO	CW
1/5/2017	51	12		12	30	Y	FO	CCW
1/11/2017	43	19		14	-30	Y	FO	CW
1/15/2017	49	18		15	30	Y	FO	CCW
1/18/2017	49	17		14	-30	Y	FO	CW
2/23/2017	42	25		12	30	Y	FO	CCW
2/24/2017	43	25		10	30	Y	FO	CCW
3/1/2017	31	27		8	-30	Y	FO	CW
3/14/2017	46	18		8	-30	Y	FO	CW
3/16/2017	38	16		10	30	Y	FO	CCW
3/29/2017	38	12		8	-30	Y	FO	CW
3/30/2017	43	24		15	30	Y	FO	CCW
4/4/2017	38	15		3	-30	Y	FO	CW
4/6/2017	39	13		6	30	Y	FO	CCW
4/7/2017	30	17		4	-30	Y	FO	CW
4/12/2017	31	13		10	30	Y	FO	CCW
4/13/2017	35	13		5	-30	Y	FO	CW
4/18/2017	33	14		4	30	Y	FO	CCW
4/19/2017	39	17		3	-30	Y	FO	CW
4/20/2017	36	17		6	30	Y	FO	CCW
4/26/2017	39	17		11	-30	Y	FO	CW
4/27/2017	37	22	10		30	Y	FP	CCW
5/10/2017	35	11	11		-30	Y	FP	CW
5/11/2017	36	10	15		30	Y	FP	CCW
5/12/2017	42	26	20		-30	Y	FP	CW
5/16/2017	41	24	18		30	Y	FP	CCW
5/17/2017	39	13	13		-30	Y	FP	CW
5/19/2017	35	21	9		30	Y	FP	CCW
5/31/2017	39	28	6		-30	Y	FP	CW
6/1/2017	39	9	7		30	Y	FP	CCW
6/9/2017	39	14	12		-30	Y	FP	CW
6/15/2017	49	3	10		30	Y	FP	CCW
12/5/2019	58	36		55	30	Z	FO	CCW
12/6/2019	68	60		77	30	Z	FO	CCW
12/7/2019	55	32		59	30	Z	FO	CCW
12/9/2019	71	37		67	30	Z	FO	CCW
12/12/2019	56	44		56	30	Z	FO	CCW
12/14/2019	57	56		60	30	Z	FO	CCW

1/10/2020	50	56		48	30	Z	FO	CCW
1/21/2020	50	67	26		30	Z	FP	CCW
1/22/2020	49	59	37		30	Z	FP	CCW
1/23/2020	50	65	32		30	Z	FP	CCW
1/24/2020	50	59	39		30	Z	FP	CCW
1/25/2020	65	62	36		30	Z	FP	CCW
1/28/2020	57	50		30	30	Z	FO	CCW
1/29/2020	49	43		37	30	Z	FO	CCW
1/31/2020	53	42		39	30	Z	FO	CCW
