# Supplementary material for: Frontal and parietal planning signals encode adapted motor commands when learning to control a brain–computer interface
Source: PLoS Biol. 2025 Sep 29;23(9):e3003408. doi: 10.1371/journal.pbio.3003408 (PMC12533969; doi:10.1371/journal.pbio.3003408)
Supplement: Attachment — Submitted filename: Answer_to_reviewers.pdf [file pbio.3003408.s022.pdf]

Editor

Another concern that we would like to highlight regards the imbalance in data between the two monkeys. In several cases, Monkey Z appears to have very few data points (for example, one t-test has df = 4, and another has df = 9), raising questions about the robustness of the data analysis and the strength of the overall conclusions, given that the study is based on only two monkeys.

Using two animals is accepted standard in the field, acknowledging the ethical need for keeping the number of animals as small as possible. Statistics in this case do not built on the N=2, i.e., the do not make an inference on the population on animals, but rather on the within-animal testing. Please also note that the experimental condition/animal in which the statistical power is limited due to small number of repetitions, is the case with higher effect size, yielding significance despite small sample size. Experiments in this animal were terminated early because implants failed, while being agnostic about the results. We consider our conclusions conservative and justified given that our statistical tests showed significant results besides limited sample sizes and due to the fact that the conditions with comparable sample sizes also show consistent findings between both animals.

Reviewer #1:

In this study, Gail's lab investigated the frame of reference for motor planning and execution in reaching using a brain-computer interface (BCI) paradigm. The study involved the primary motor cortex (M1), dorsal premotor cortex (PMd), and the parietal reach region (PRR).

The main finding is that both frontal and parietal regions reflected adapted motor commands more effectively than perturbed visual feedback during both movement preparation and execution. PRR exhibited motor-like encoding during learning, even when it was not directly controlling the movement, suggesting that PRR plays a more integral role in motor adaptation than merely reflecting visual feedback. However, the transfer from planning to movement was stronger in the frontal cortex compared to PRR.

This manuscript clearly demonstrates that both the frontal and parietal cortices encode reaching in a motor-reference frame, indicating a re-aiming mechanism for both cortices. This process occurs during both the planning and execution phases of reaching. The manuscript is well-structured, with clear hypotheses and well-designed methods to test them. The presentation is highly effective, with well-crafted figures that guide the reader through the reasoning (e.g., Figure 3).

I particularly appreciated the paradigm, which studies reaching movements in 3D rather

than the conventional center-out task. In this paradigm, the BCI approach ensures constant proprioceptive feedback, making the task reliant solely on visual and motor signals. While prior work by the Batista lab demonstrated the preservation of the neural manifold in the M1-PMd cortex during short-term adaptation, this manuscript extends that concept to include BCI non-controlling units and PRR. This work is thus significant in extending the understanding of motor adaptation to both the parietal cortex and frontal neurons not driving the BCI.
Major Points:

1. M1 and PMd differentiation: It would be valuable to separate the neuronal pools of M1 and PMd and verify whether the results hold true for each region independently.

The limited number of cells per each area limited the precision of the reconstructed trajectories when conducting the decoding analysis in M1 and PMd separately. We still assessed the individual contributions of each area with the help of single unit tuning analyses. We computed each neuron's preferred direction and performed separate statistical analyses for the two populations. This approach has also been used in previous literature as an effective measure of neural shift (Chase et al., 2012; Jarosiewicz et al., 2008; Vyas et al., 2018)

For this analysis, we compared the preferred direction angle for each neuron in the frontoparallel plane (i.e., the plane of the applied perturbation) between baseline and late rotation trials, following the method described by Jarosiewicz et al. 2008 for the 3D case. We consistently observed a shift in the preferred direction of cells in both M1 and PMd, aligning with the direction of the trajectory suited to counteract the applied perturbation.

We now include these data in a new supplementary figure S4 and refer to it in lines 299-302 in the main manuscript.

2. PRR targeting: Please clarify which specific area of PRR was targeted, considering the depth of the recording electrodes. For Monkey Z, in the supplementary material, include the position of the interhemispheric midline to better evaluate which portion of the posterior parietal cortex (PPC) was studied.

We expanded the respective paragraph in Methods (lines 667-677) and updated supplementary Fig. S1 accordingly. We now provide more detailed stereotactic position information and explain that, given the laterality of the parietal arrays, we targeted area MIP, not V6A. Due to the length of few shorter electrodes (1.9 mm up to 7.1 mm), we likely also sampled from the more superficial area 5. We added the photo image for animal Z and provide now schematic drawings for both animals, to contain information about the position of the midline and the coordinates for each array.

3. Frame of reference for reaching: On line 84, it is stated that "Preparatory activities include reach-goal information in different body-related frames of reference, including the predominantly gaze-centered parietal reach region (PRR)." Since different regions within the PRR area have been shown to employ various reference frames for reaching, this alternative version should also be mentioned (e.g., Bosco et al., 2016, Scientific Reports; Piserchia et al., 2017, Cerebral Cortex; Hadjidimitrakis et al., 2020, Journal of Comparative Neurology).

We thank the reviewer for the valuable hint. We expanded the introduction paragraph (lines 95-105) accordingly to broaden the view and take into consideration also neighboring areas in posterior parietal cortex.

4. Discussion - 3D reaches: At the beginning of the discussion, the authors highlight the importance of studying 3D reaches rather than 2D movements. It would be appropriate to cite prior work from other groups that studied 3D reaches (e.g., Fattori lab in PPC, Filippini et al., 2020, Journal of Neural Engineering; Hadjidimitrakis et al., 2022, Cell Reports).

While the 3D representations are not in the focus of our three main research questions, and hence also not our discussion and conclusions, we agree that previous related work deserves mentioning for a more comprehensive picture. We added suggested recent and also earlier studies to the Discussion section (lines 496-499).

5. PRR decoding of motor plans: Regarding lines 290-300, some clarifications are needed:

- How was the low yield in PRR units mitigated?
- What does the statement "the controlling PRR neurons were combined with a subset of M1-PMd units in the FP decoder sessions" mean? Why was this necessary?
- How many units were recorded in PRR, and what was the threshold needed for statistical significance? What were the yields for M1 and PMd?
- The fact that in PRR we see similar "motor" characteristics may be it caused by the M1-PMd contamination (FP decoder session M1-PMd units added to PRR, lines 289-294)?

While the yield of units in PRR was lower than the combined yield in M1 and PMd, the main limitation for achieving effective BCI control with only PRR units was not the yield itself, but rather that the area seems suboptimal for this purpose in our experiments. We now include a discussion of these findings (lines 324-326).

Regarding yield, we reported the number of cells recorded per each experimental session in Supplementary Table 1.

To rule out the possibility that decoding performance in the full-population (fronto-parietal: FP) session was driven by contamination from M1/PMd units, we had retrained the decoder offline using only PRR units. To further ensure that PRR was not simply inactive or overridden by M1/PMd contributions, we had analyzed the velocity vector amplitudes decoded from PRR alone. As shown in Figure 4A, there was no significant difference in the amplitude of the velocity vector compared to the full-population decoder, indicating that the contribution to decoding was equally distributed across areas. In Figure 4B, we show the firing rate of PRR units during decoding, further confirming that PRR units were active and contributed meaningfully and this type of analysis consider its contribution independently of M1-PMd cells.

To make this rationale easier accessible to the reader we added the following to the manuscript (line 324-326):

"This design choice was motivated by the fact that we observed lower performance in pre-testing trials when using PRR alone, possibly due to its weaker motor coding properties"

6. Working space limitation: The 3D reaching cube was 7 cm on each side, which does not encompass most of the working space. Was this small size chosen due to limitations in covering the peripersonal space? Please provide some insight and include a comment in the manuscript about the implications of this limited working space on the generalizability of the conclusions.

Our setup utilizes dichoptic viewing for stereoscopic depth information about the 3D cursor position. This enables the animal to perform 3D reaches using a brain-controlled cursor, a challenging brain-computer interface (BCI) task with a limited amount of 50–60 units per session. Limiting the workspace size was beneficial for achieving high accuracy and keeping the animal engaged for a sufficiently high number of trials that was needed within each session to go through all phases of the adaptation paradigm (BCI training, baseline, adaptation, re-adaptation).

.

7. Perturbations: In lines 177-180, please justify why perturbations were not applied in the Z dimension (depth).

We acknowledge that studying more complex perturbations would have made our study potentially even more insightful, as such perturbations are rarely explored in psychophysical settings. In designing this study, we leveraged preliminary findings from our lab (Ferrea et al., 2022), where we observed that perturbations in the fronto-parallel

plane are significantly easier to learn compared to those in other planes. Based on this, we opted to begin with a simpler setting while maintaining the goal of incorporating more complex perturbations in future studies. We add this explanation now to the Introduction section (lines 202-204).

Moreover, while not considering it likely, we do not rule out the possibility that adaptation may occur outside the same neural manifold if depth perturbations are included (see lines 492-496 in Discussion). Such differential findings would have important implications for understanding the flexibility of neural representations in motor learning but go beyond the scope of the current study.

8. Results (lines 279-280): If I understand correctly, the non-controlling neural population adapts to the VMR as much as the controlling population for Monkey Z (94%, with 100% indicating the same level of adaptation). However, for Monkey Y, this measure drops to 59%. This discrepancy suggests a significant difference in the adaptation process between the two animals. Could the authors compute a null distribution or reference values? What is their interpretation of this difference?

We presented the adaptation results of the non-controlling units in two complementary ways: first, as the deviation in millimeters along the perturbation axis (absolut value), and second, as a scaled version of the angular deviation relative to the final adaptation level of the controlling units (relative value).

Absolute adaptation levels are comparable between both animals, demonstrating that non-controlling populations are clearly and highly significantly influenced by the perturbation in both animals (Fig. 3C). Relative modulation of the non-controlling with 94% in Monkey Z and 59% in Monkey Y indeed seem to differ to a certain degree, as the reviewer correctly points out, suggesting that the overall higher level of adaptation in monkey Y (Fig. 2D) is only partially reflected by the non-controlling units in this animal. We could speculate that this discrepancy reflects inter-individual differences in how adaptation is distributed across neural populations. Yet, at the same time, the neural tuning shifts are highly similar between controlling and non-controlling units in both animals (new Supplementary Fig. S4).

9. BCI unit selection: The authors selected subsets of neurons to drive the BCI, using either frontal-only (FO) or mixed frontal-parietal (FP) units. While I assume this was done for performance reasons or due to limited motor coding in PRR, this rationale should be clearly stated. Lines 289-292 seem to address this but are somewhat unclear.

We have now added a clarification to explain why this was necessary. As the reviewer correctly points out, control by the PRR alone showed reduced performance during pre-tests, which we did not want to combine with experimentally induced perturbation to keep the animals motivated and engaged (lines 324-326).

Minor Points:

1. Introduction (lines 83-91): This section seems confusing, possibly due to typos from older manuscript versions.

Thanks for pointing this out, we now improved readability to avoid confusion.

2. Results (lines 337-339): Include numerical results (or graphs) in the main text to support these claims.

We now included numerical results in the main text as suggested (lines 364-367).

3. Figure S5 legend: Add more detailed information to help readers better understand the data presented.

We now extended the legend (now figure S6) to better clarify the method.

Reviewer #2:

Ferra and colleagues present new BCI experiments to study how frontal and parietal motor areas contribute to short term decoder perturbations. Their study incorporates three new aspects that allow them to expand upon prior work examining adaptation to rotational-perturbations in BCIs:

1) recording from parietal regions,

2) comparing adaptation in the neural populations that drive the BCI ("controlling") and those that do not ("non-controlling"), and

3) using a delayed reaching task to examine planning activity. The paper generally presents clear data that support the hypothesis that frontal and parietal areas both contribute to adaptation mechanisms, and that neural activity patterns are consistent with a "re-aiming" strategy that occurs during planning phases. The work includes many challenging experiments and thorough analyses that are to be commended, and contributes to an active literature discussing learning mechanisms in BCI.

I have a few more major concerns:

1) The paper is currently written to test core predictions of the hypothesis that rapid visuomotor rotations are learned via "re-aiming" in BCI. This hypothesis is a predominant view within the many recent BCI learning studies. While further supporting established hypotheses is not a weakness fundamentally, the paper may benefit from framing the contributions to better center the novel contributions of the work.

We thank the reviewer for pointing to the important question of how to frame the research goal in order to ensure that the novel aspects become sufficiently clear. In the revised title, we highlight the large-scale neural adaptation across frontal an parietal regions and the fact that this adaptation happens in a motor reference frame in both regions. In the abtract, we further point to the fact that the relationship between movement planning and execution changes over the course of learning. All three findings are new and we are not aware of previous such comparisons in a BCI learning paradigm, nor in in classical VMR tasks. It is non-trivial that parietal representations adapt in a motor reference frame, even if assuming a re-association mechanism. Consequently, we show the result of stable network structure (subspace alignment across adaptation phases) in support of the re-association model in the Supplemental Materials only (Supplementary Fig. S2 and S3), and focus on the three novel research questions in the main text. In the Introduction and Discussion sections, we still felt that we should discuss the state-of-the art with respect to rapid adaptation and the concept of “re-association” since it is a prominent concept, as the reviewer also points out. We carefully checked the phrasing throughout the manuscript to avoid the impression that “re-association” versus “reconfiguration” itself was the main focus of the study (see the reply to the following question regarding the terms re-aiming versus re-association).

2) An inter-related comment: The manipulations performed to allow the researchers to analyze planning activity (introducing a delay period task) may influence their findings confirming a "re-aiming" hypothesis. Past work in human psychophysics highlights a relationship between the time people have to prepare and the expression of "explicit" motor learning strategies such as re-aiming (e.g., Haith et al., J Neurosci 2015; Leow et al., J Neurophysiol 2017). Indeed, Leow et al. 2017 uses delay-time manipulations to isolate implicit vs. explicit contributions to visuomotor adaptation. By introducing a long delay period, the task design may increase the animals' ability to/likelihood to use explicit strategies like re-aiming. This does not refute the paper's claims, but does add important nuance to the interpretation of the results. In particular, the likely link between the task design and learning mechanisms, in my opinion, reduces the impact of the author's claims to test
their central hypothesis about re-aiming.

The reviewer raises an important point and we are grateful for the valuable hints to the previous psychophysics literature. When making reference to the previously introduced terms “re-aiming” and “re-association”, we treated them as synonymously, as other previous work suggested. This might not be fully justified. Our animal experiment does not allow us to easily infer the cognitive state of awareness, and hence to imply an “explicit” strategy (a discussion which even for human adaptation data is multifaceted and seems controversial in parts). In hindsight, we prefer to stick with the term “re-association” used in previous physiology studies and which – according to our understanding – has less strong implications in terms of “explicitness” of the animals behavior, while still containing the idea of making use of an existing repertoire of neuro-cognitive states, no matter if a subject is aware of it or not. We revised the manuscript accordingly and only use the term re-association now. We also adapted a paragraph in the Discussion section to briefly link to previous findings about implicit/explicit adaptation.

3) One of the authors' notable (and interesting) claims is that planning activity and motor activity become more correlated with each other with learning. However, the presented analyses make it a bit difficult to fully appreciate and understand this effect. The analyses of planning and movement relationships are entirely restricted to the linear interaction model and a bar graph of coefficients. I'm a bit unclear on why, for instance, a plot similar to Fig S6 isn't included for the BCI data. The inclusion of the many possible contributing factors and interaction terms also makes it a bit challenging to interpret. I also struggle to fully understand the concluding sentence of that section ("This suggests that, over time, the same degree of re-aiming during the planning phase results in more pronounced motor corrections (data not shown)."

Thank you for giving us the opportunity to improve the accessibility of these results for the reader. To quantify how the relationship between planning-related and movement-related activity evolves during learning, we fitted a linear model. A significant slope indicates a relationship (correlation). Additionally, our linear model included an interaction term with trial number. A significant interaction (β interaction = ..., $p < 0.001$) indicates that the slope changes, i.e., the correlation between planning and movement activity changes as training progresses.

To visualize this effect, we added a new panel analogous to Fig. S6 that contrasts the regression slopes for the early (first half) and late (second half) learning phases. As visible in the figure, the slope becomes steeper in the late phase. With a steeper slope, deviations along the planning axis predict comparably larger deviations along the movement axis. One way to interpret this would be to say that by the end of the adaptation period the system achieves comparable performance with less planning effort. We added the figure and present these results better at lines 438-451.

Specific modest/minor comments:

The paper neglects to include the N for statistical tests. Please update to include these.

We now explicitly state that our reported degrees of freedom (DOF) corresponds to N-1. Lines (xxx).

The methods presented to describe how the authors analyzed each neuron's contribution to the BCI is insufficient. The authors say the used the "matrix from the Kalman Filter" but the Kalman Filter has multiple possible matrices that could be used for these calculations (the Kalman Gain matrix or the Observation model).

Thank you for spotting this ambiguity. We have now clarified in the revised Methods that we use the observation/encoding matrix and not the time-varying Kalman gain to quantify each neuron's contribution. Because the observation matrix encodes the only time-invariant link from each neuron's activity to the decoded state, it provides a stable and interpretable measure of potential influence. By contrast, the Kalman Gain merely scales the same prediction errors according to the decoder's momentary uncertainty and therefore cannot serve as a single, session-wide importance metric. We added this information at lines 747-754.

It's unclear to me that the authors conclusively rule out the possibility that the animals are trying to move during the planning phase with the current analyses. The current analyses show that the planning and movement PCs are not aligned during arm movements, and they see the same pattern during BCI. But there are aspects of the task that differ between the planning and movement phase that may contribute to this misalignment, such as the animal being allowed to release fixation. That transition is the same between BCI and the arm movement task. If those task-related factors are the primary driver of the alignment change - as opposed to the transition from planning to movement - this test would not reveal whether or not the animals are attempting to move in BCI.

We apologize if the arguments in this paragraph were not apparent. Our planning-movement relationships in state space align closely with analogous findings from motor cortex using similar memory-guided reach tasks with free gaze (Ames et al., 2014; Vyas et al., 2018) but we agree that eye fixation compared to free gaze paradigms can show distinctly different neural activity (Batista et al., 2007; Boussaoud et al., 1998).Moreover, if fixation release was the dominant factor explaining the misaligned

state spaces, we would expect faster change in the population activity, beginning as soon as the central fixation cue disappeared We are convinced that the animals did not attempt to move the cursor during planning in BCI control since the neural response onset latencies in response to the go-cue were highly similar between BCI and manual trials. This would not be the case if the movement command would already be produced during delay and then immediately released with the go-cue. We adjusted the text accordingly at lines 371-376.

The discussion paragraph related to differences between rule-based learning in discrete BCIs and the learning observed in this task is a bit unclear. The exact distinction the authors aim to draw between "rule-based" and explicit/implicit is not clear. I find this section particularly confusing in light of my comments above about how even "continuous" VMR tasks can use a mixture of mechanisms depending on the task structure.

We thank the reviewer for this insightful comment and have revised the Discussion to better clarify the distinction between rule-based (explicit) and implicit learning mechanisms (see lines 515–534), although we tend to be cautious about implying explicit strategies in animals. A recent study from our lab discusses this in more detail (Guo et al., 2025).

I am not clear I understand what aspects of their findings the authors feel support the claims that: "...the adaptive mechanism became more efficient over the course of learning" and "During the later stages of adaptation, it appears that less re-aiming is required during planning..." (discussion)

Thank you for pointing out that this part seems not to have been clear enough. We now added new panels in figure 6. We tried to explain better in the discussion that a steeper relationship between the early and late phases of adaptation implicate a higher level of movement correction (lines 438-451).

In the intro: "fast adaptation, which is particularly desirable for BCI learning". Desirable by who? For BCI applications? What exactly do the authors mean by this? It is unclear what link the authors see between adaptation to artificially-introduced perturbations and learning that would occur in clinical BCIs, for instance.

What we meant here is that when subjects have to learn new control of a BCI cursor, it is advantageous to have faster learning processes to improve control with fewer learning demands. We thank the reviewer for highlighting that our original sentence might not have been clear for the audience, and we have adjusted it accordingly. (lines 79-81)

The authors use the term "co-adaptation" to refer to learning mechanisms that seem to co-occur. Within the BCI field, the term "co-adaptation" is often used to refer to learning of the decoder and the brain (e.g., Taylor et al., Science 2003). The authors may benefit from choosing a term that is not already "loaded" with preconceptions for certain audiences.

We used now the term distributed adaptation all over the manuscript, to avoid confusion.

Line 260: "As a result," phrasing is a bit confusing. Do you mean to highlight that this is a result you found?

We adjusted to “in our results” to highlight the fact that this comes from our analysis. (line 289)

The paper cites Jiang et al., 2020 within the manuscript but this is not included in the bibliography. This omission is particularly important since you're referring to a method, which I couldn't evaluate.

Thanks for pointing this out, we corrected it.

I would suggest that the statistical analyses/results presented in lines 305 - 319 could likely be presented much more concisely/efficiently.

We now shortened and compacted the description as suggested (now lines 339-343)

The discussion states "This within-manifold adaptation suggests that functional connectivity remains relatively unchanged during VMR learning." None of the cited papers, however, directly prove this claim. All experimental papers cited do not analyze/measure functional connectivity. The modeling paper does make predictions about what connectivity changes do/don't need to change, but one key observation of that paper is that there is more nuance to the interpretation of the in/out of manifold BCI learning and the link between network connections (since it could also be explained by feedback available to guide learning in the RNN). Please revise.

We adjusted the terminology and used the more subtle term “constraints imposed by the underlying neural circuitry” as also referred in the previous literature.

The supplemental table of experimental sessions could benefit from some formatting revisions to make it easier to read (e.g., the alignment is inconsistent across rows; it's not always clear what number goes with which column label due to alignment).

Thank you for catching this. We reformatted the table for better readability.

References

Ames, K.C., Ryu, S.I., Shenoy, K.V., 2014. Neural dynamics of reaching following incorrect or absent motor preparation. Neuron 81, 438–451.

Batista, A.P., Santhanam, G., Yu, B.M., Ryu, S.I., Afshar, A., Shenoy, K.V., 2007. Reference Frames for Reach Planning in Macaque Dorsal Premotor Cortex. Journal of Neurophysiology 98, 966–983. https://doi.org/10.1152/jn.00421.2006

Boussaoud, D., Jouffrais, C., Bremmer, F., 1998. Eye Position Effects on the Neuronal Activity of Dorsal Premotor Cortex in the Macaque Monkey. Journal of Neurophysiology 80, 1132–1150. https://doi.org/10.1152/jn.1998.80.3.1132

Chase, S.M., Kass, R.E., Schwartz, A.B., 2012. Behavioral and neural correlates of visuomotor adaptation observed through a brain-computer interface in primary motor cortex. Journal of Neurophysiology 108, 624–644. https://doi.org/10.1152/jn.00371.2011

Guo, H., Kuang, S., Gail, A., 2025. Sensorimotor environment but not task rule reconfigures population dynamics in rhesus monkey posterior parietal cortex. Nature Communications 16, 1116.

Jarosiewicz, B., Chase, S.M., Fraser, G.W., Velliste, M., Kass, R.E., Schwartz, A.B., 2008. Functional network reorganization during learning in a brain-computer interface paradigm. Proc. Natl. Acad. Sci. U.S.A. 105, 19486–19491. https://doi.org/10.1073/pnas.0808113105

Vyas, S., Even-Chen, N., Stavisky, S.D., Ryu, S.I., Nuyujukian, P., Shenoy, K.V., 2018. Neural population dynamics underlying motor learning transfer. Neuron 97, 1177–1186.
