# Supplementary material for: Frontal and parietal planning signals encode adapted motor commands when learning to control a brain–computer interface
Source: PLoS Biol. 2025 Sep 29;23(9):e3003408. doi: 10.1371/journal.pbio.3003408 (PMC12533969; doi:10.1371/journal.pbio.3003408)
Supplement: Attachment — Submitted filename: Answer to reviewer2.docx [file pbio.3003408.s023.docx]

Reviewer remarks:

Reviewer #1: All my suggestions have been implemented graciously.
We thank the reviewer for assessing our revision.

Reviewer #2: I thank the authors for their thoughtful revisions to the manuscript and responses to my comments. The paper is significantly improved and largely addresses my concerns. I have two lingering issues after reviewing the new manuscript:
1.      I do not feel the current discussion section adequately addresses my comments about distinctions between implicit/explicit computations. I take the authors point (in the rebuttal) that the link between these notions and "re-aiming" as discussed in the monkey/BCI literature is not yet clear. Yet, I think many studies (and, importantly, readers) often implicitly make this tempting link between the two separate lines of research. The authors seem to be arguing that explicit strategies cannot occur in continuous VMR tasks, but plenty of human psychophysics studies (e.g., the ones I cited in my prior critique) do observe a mixture of explicit and implicit strategies in continuous VMR tasks. I think it's important that this nuance and potential ambiguity is explicitly addressed in the discussion (relevant paragraphs: "In tasks that impose a rule, …" and the following one, "Our experiment differs in at least one aspect…".

We thank the reviewer for further clarifying this point. We updated our discussion, in line with the reviewer’s comment, about the implicit and explicit mechanisms which might contribute to learning in our task, also considering the effect that the delay period might have (see lines 535–546).

2.      The new manuscript includes the sentence "Instead what we observed where cursor movements and associated neural response latencies similar to manual reaches." I could not find any data provided to support this claim. Can you please clarify?

We added clarification to the main text (lines 373–380) and an additional figure panel to the supplemental materials (Figure S3-I) to better explain the rationale behind this argument.

We thanks the reviewer for the additional comments, which we truly believe have further improved our manuscript, and we hope that the current version can now be accepted for publication.
